# A comprehensive review of the childhood vaccination landscape in Malaysia

**DOI:** 10.1017/S095026882500024X

**Published:** 2025-02-20

**Authors:** Nor Kamila Kamaruzaman, Marco Rizzi, Katie Attwell

**Affiliations:** 1 VaxPolLab, School of Social Sciences, The University of Western Australia, Perth, Australia; 2 UWA Law School, The University of Western Australia, Perth, Australia

**Keywords:** vaccination (immunization), vaccine preventable diseases, vaccine hesitancy, mandatory, policy

## Abstract

Vaccination is one of the most cost-effective and successful public health interventions to prevent infectious diseases. Governments worldwide have tried to optimize vaccination coverage, including using vaccine mandates. This review of recent literature and policy aims to provide a comprehensive overview of Malaysia’s childhood vaccination landscape. The document analysis was used to identify and examine information from government policy documents, official government media statements, mainstream news content, and research papers. Content analysis was then employed to analyze the gathered information. Despite the successes of Malaysia’s National Immunization Programme, a resurgence of vaccine-preventable diseases has raised concerns about vaccine hesitancy and refusal. Several contributing factors have been identified, including a preference for alternative medicines, doubts about halal status, fear of vaccine injury, concerns about the vaccines’ contents, conspiracy theories, as well as convenience and access barriers. While various initiatives have been implemented, Malaysia may consider using vaccine mandates, as several countries have recently done, as a potential policy intervention to address these challenges. This review benefits policymakers, epidemiologists, as well as researchers involved in regional or global policy planning and advocacy efforts. It also offers comprehensive insights into designing effective interventions and making informed policy decisions regarding childhood vaccination programmes.

## Introduction

In 1974, the World Health Organization (WHO) launched the Expanded Programme on Immunization to ensure vaccine access for all children regardless of socioeconomic status or geographical barriers. Since then, multifaceted strategies have been developed, and today, every country has established national immunization programmes (NIPs) [1]. High vaccination coverage helps prevent the spread of vaccine-preventable diseases and also positively impacts health system efficiency and economic growth. Hence, governments around the globe strive to encourage the uptake of available vaccines using persuasion and education campaigns as well as – in some cases – coercive methods via mandates to achieve or maintain community protection [2]. One hundred and five countries had national vaccination mandates as of December 2018 [3]. MacDonald et al. (2018) [4] discussed that among the factors leading to coercive methods was the perceived failure of ‘soft’ modes of governance such as ‘persuasion and nudging’. Recent outbreaks of measles and polio have also prompted calls for mandates as this is considered a straightforward solution to address sub-optimal uptake of vaccines [4], with Vanderslott and Marks [5] suggesting that mandatory childhood vaccination is becoming a crucial policy intervention in public health nowadays to achieve high vaccination rates.

However, scholars and international organizations note the importance of addressing barriers to accessing health systems by providing vaccines free of charge, optimizing service experience, and ensuring that vaccine encounters are culturally appropriate for diverse communities – these measures should be in place before authorities consider coercive policies [4, 6]. Public communication strategies and funding the training of health professionals to counsel hesitant parents are further interventions that should precede mandates [7]. An experimental study by Betsch and Böhm [8] found that mandates may also be risky because they can generate opposition from the population. In light of ethical considerations, scholars also suggest mandates be the last resort to counter vaccine refusal [8, 9]. Even so, some countries in the Global North successfully implemented coercive policies to increase vaccination coverage [10]; doing so in Malaysia may have different implications and would need careful local examination of the factors that would contribute to such a policy’s success [11].

In the context of Malaysia, the only recent discussion about the comprehensive NIP has been in the form of a book launched by the Ministry of Health (MOH). This non-academic publication provides insights with regard to its past, present, and future [12]. Furthermore, only two presentation papers by Ja’afar [13] and Kusnin [14] at international symposia highlighted childhood vaccination in Malaysia, and no recent academic articles provide a comprehensive overview of the topic. Sustainable Development Goal (SDG) 3 aims to eliminate preventable deaths of children under five by 2030 [15]. According to the United Nations International Children’s Emergency Fund (UNICEF) [16], many children are still dying, underscoring the need for concerted efforts from various bodies to ensure the health and well-being of children. Note that similar challenges may be present in other countries, particularly those with sociodemographic characteristics akin to Malaysia, such as Indonesia [17, 18] or Muslim-majority countries like Pakistan [19].

Therefore, this review provides an up-to-date “lay of the land” of Malaysian childhood immunization policy and practice. This review would also offer strategies to address similar challenges in those countries with sociodemographic characteristics similar to Malaysia. Moreover, insights from this review could significantly provide information for policymakers, epidemiologists, as well as researchers at regional and global levels in designing effective interventions and making informed decisions to prevent infectious diseases among children. Ultimately, the outcomes of this review will contribute to achieving the targets of SDG 3.

As such, this article is structured as follows. The next section describes the review methodology in general. The results section provides a comprehensive description of Malaysia’s NIP, describes issues and challenges for vaccination in Malaysia as well as initiatives taken by the MOH to ensure access and vaccine acceptance throughout the country, and highlights future perspectives for Malaysia, including the possible implementation of vaccine mandates to address the challenges.

## Methods

This article presents a narrative review approach to capture a broader range of documents, as suggested by Greenhalgh et al. [20]. Document analysis is a valuable research method to examine various types of documents containing text, including institutional reports, books, journal articles, and newspaper articles [21]. First of all, the authors identified and examined Malaysian government policy documents, institutional reports, official government media statements, and mainstream news content available online between 1 August 2023 and 15 March 2024 without imposing restrictions on the time frame of the documents. Factors including authenticity, credibility, and meaning by aiming for primary data sources and reliable sources, such as government official websites, were considered to ensure the reliability and authenticity of the included materials [22]. Next, to identify contributing factors of vaccine hesitancy and refusal in the Malaysian context, articles in the Google Scholar database were searched using the main keywords: “vaccine hesitancy” OR “vaccine refusal” AND “childhood” OR “children” AND “Malaysia.” In particular, relevant articles published from 2016 until 2023 were selected for this screening stage. Instead of just relying on the keyword search, a snowball technique from the seed articles was also adopted to produce a network of relevant articles. Researchers from various disciplines widely use content analysis, as this approach aims to provide a representation of facts, new insights, knowledge, and a possible guide to action [23]. In this regard, deductive content analysis, a top-down approach, was employed to code the data theoretically using pre-existing concepts from the literature review [24], hence elucidating a comprehensive overview of the childhood vaccination landscape in Malaysia.

## Results

### Childhood immunization programme in Malaysia: evolution, policies, budget, vaccine safety and service delivery

**Figure 1.**
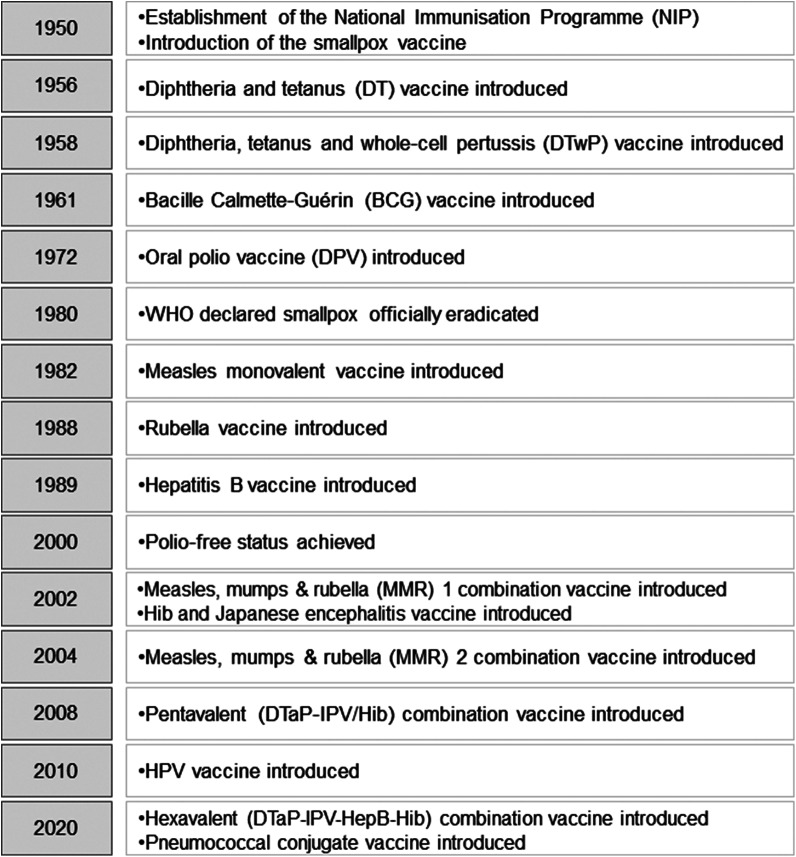
The history and evolution of the NIP [12].

**Figure 2.**
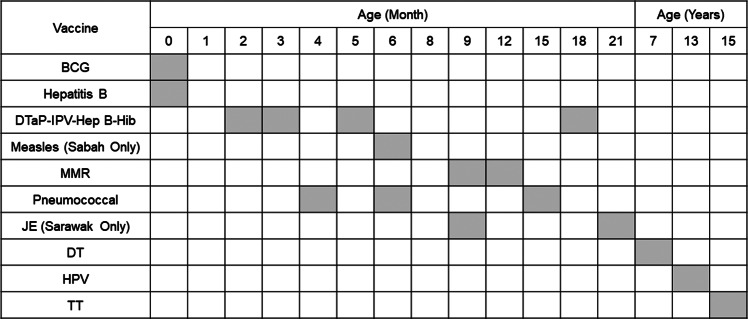
The National Immunization Schedule [26].

Malaysia’s NIP was established in 1950 with the introduction of smallpox vaccine. Vaccination is free under the NIP for all children under the age of 15 years; since 2015, non-citizens have to pay a small fee [14]. The NIP protects children from 13 vaccine-preventable diseases, namely measles, mumps, rubella, diphtheria, pertussis, tetanus, Japanese encephalitis, polio, tuberculosis, *Haemophilus influenza* type B, human papillomavirus, hepatitis B and pneumococcal [12]. Meanwhile, additional recommended vaccines, like rotavirus, influenza, and varicella, are available at private facilities for some fee [25]. Drawing from existing literature, Figure 1 illustrates the history and evolution of the NIP, and Figure 2 shows the current national immunization schedule in Malaysia.

In its 10-year strategy for child health published in 2021, the MOH reported high coverage for all childhood vaccinations, with more than 95% achieved apart from a slight decrease in the MMR uptake rate in 2014 and 2015 [27]. It is rare to encounter neonatal tetanus and *Haemophilus influenza* type B nowadays [12]. However, the Institute for Public Health revealed that the completed primary vaccination uptake for children under 2 years old has significantly dropped from 95.3% in 2016 to 87.1% in 2022. The reported justification was the COVID-19 pandemic and the movement control order that limited citizens’ access to healthcare [28], but there may be other drivers.

The WHO has outlined National Immunization Technical Advisory Groups (NITAGs) as crucial components of a country’s immunization system. NITAGs are composed of experts from various fields who are responsible for providing evidence-based recommendations to the government on policy issues related to immunization and vaccines [29]. Malaysia has established a NITAG [30]: the National Immunization Policy and Practice Committee, which is the highest level of technical and advisory working group on immunization and vaccines. It is chaired by the Director-General of Health and comprises relevant stakeholders, experts, paediatric consultants as well as the Malaysian Paediatric Association. This committee acts as the decision-making body for policies and strategies of the NIP [12].

**Figure 3.**
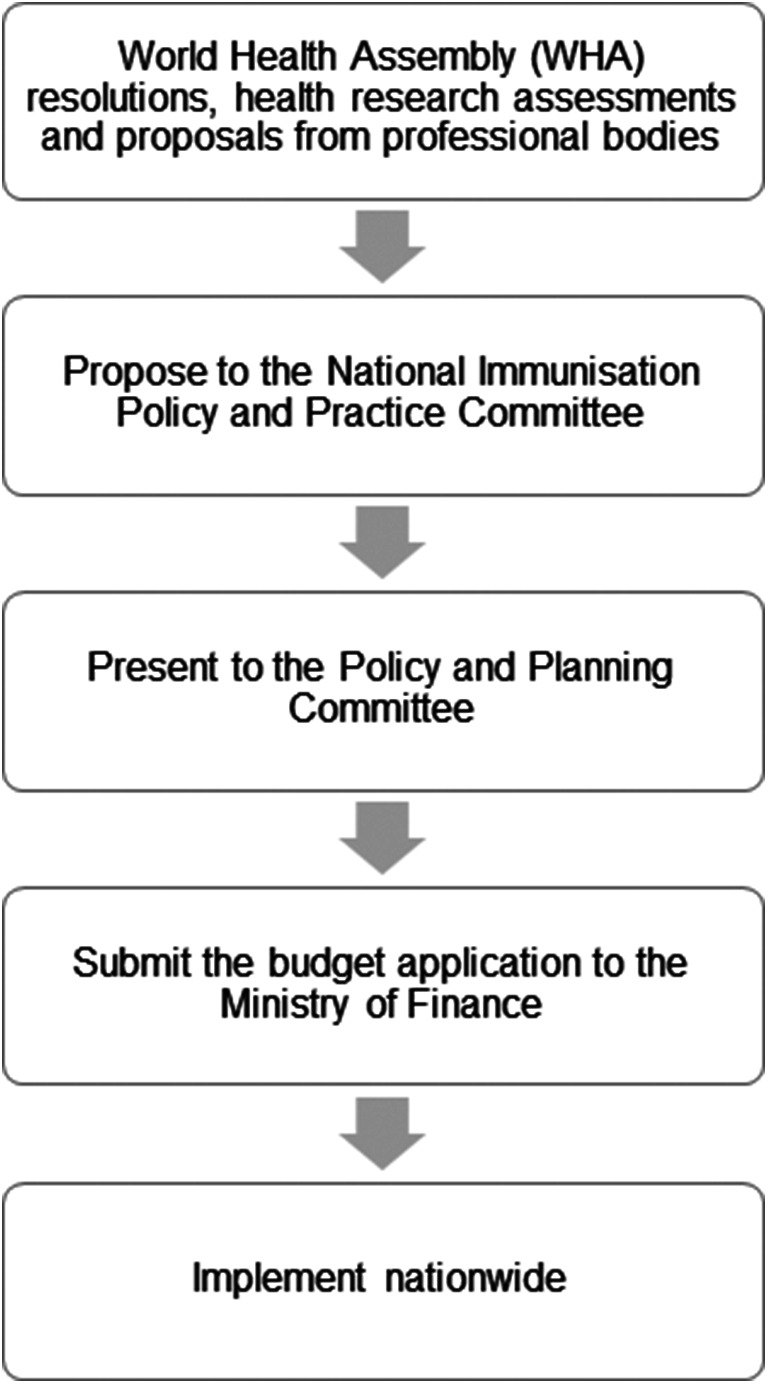
Process flow on introducing the new vaccine in Malaysia [13].

New policies and strategies regarding immunization in Malaysia are contributed by several sources such as World Health Assembly resolutions, health research assessments, and proposals from professional bodies [31]. The MOH further elaborates that immunization policies are developed by taking into consideration parameters such as morbidity, mortality, hospitalization, outbreaks, and local data from neighbouring countries. Moreover, cost-benefit analyses are also conducted to evaluate the feasibility of implementing such policies [12]. The committees involved in the introduction of new vaccines are (1) pharmacovigilance on safety of vaccines; (2) immunization implementation; (3) vaccine use and cost; and (4) health education and promotion. The recommendation of new vaccines is proposed to the National Immunization Policy and Practice Committee. If they agree, it is then presented to the Policy and Planning Committee, which is co-chaired by the Secretary General of Health and the Director-General of Health. After that, the MOH submits the budget application to the Ministry of Finance before nationwide implementation [13]. Drawing from the literature, the process flow of introducing a new vaccine in Malaysia is summarized in Figure 3. Despite a recent rise in vaccine cost per child from approximately RM327 (USD$68.65) in 2015 to RM564 (USD$118.40) in 2020, the NIP obtains a significant budget every year from the Ministry of Finance to ensure the continuous supply of vaccines. The budget also considers training to ensure staff are provided with the required skills, such as handling and administering vaccines properly as well as communicating effectively with parents [12].

The MOH prioritizes the safety, quality, and efficacy of vaccines available in Malaysia. The National Pharmaceutical Regulatory Agency (NPRA) is responsible for pre- and post-licencing all vaccines in Malaysia. Manufacturers or applicants must submit a dossier during pre-licencing for a series of assessments. Post-licencing is related to post-marketing surveillance in which the NPRA will perform vaccine lot release evaluation, cold chain inspection, and physical appearance test for every imported vaccine lot. The NPRA also monitors reports on adverse events following immunization (AEFI) by consumers or healthcare professionals after the vaccine is available in the local market [12].

**Figure 4.**
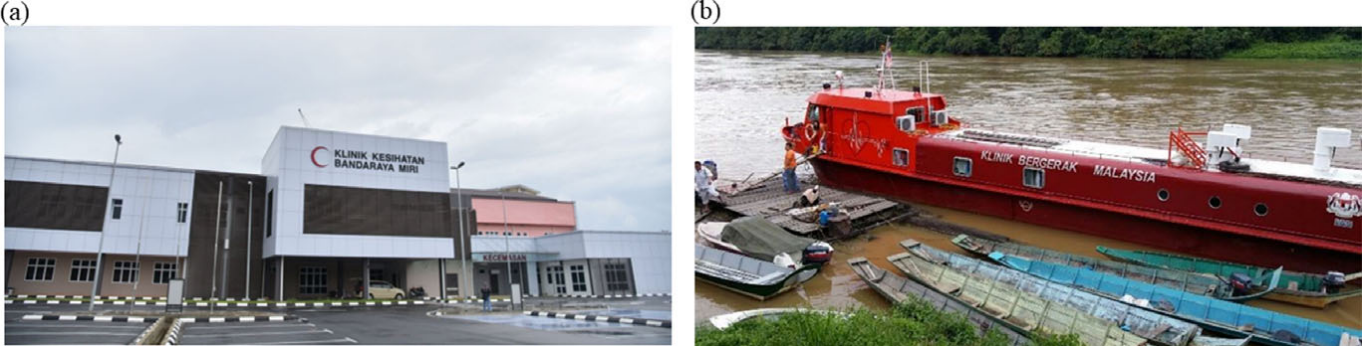
(a) Government clinics in Malaysia [32]. (b) Riverine mobile clinic for remote areas [33].

Malaysia has one government clinic every five square kilometres. Most of the population lives within that radius (as shown in Figure 4a), facilitating parents to get free immunization easily for their children [12]. Some vaccines are also delivered through the school health service programme via a mobile team [14]. Apart from collaborating with the Ministry of Education, the MOH also works closely with the Department of *Orang Asli* (Indigenous) Development and non-governmental organizations to provide immunization to marginalized groups. In Malaysia, many *Orang Asli* live in rural areas or deep in the jungles. To ensure they are not left behind due to geographical barriers, the MOH provides health services via a flying doctor team to remote areas. The MOH is also committed to ensure vaccine access by providing riverine (as shown in Figure 4b) and sea mobile clinics for communities in the remote areas of Sabah and Sarawak. From time to time, the MOH will also conduct supplementary immunization activity by visiting the homes of those who miss out on their vaccinations [12].

### Challenges for vaccination in malaysia

The resurgence of vaccine-preventable childhood diseases, especially measles, followed by several deaths from diphtheria and pertussis, has raised concern about the possible rise in vaccine hesitancy in Malaysia [34]. Based on the data collected from government health facilities, the MOH identified that the number of Malaysian parents who refuse to vaccinate their children increased from 637 in 2013 to 1603 in 2016. As data were derived from government health facilities only, the actual figures might be higher as input from private clinics was not included [12]. In general, the highest figures for vaccine refusal were reported in Kedah, Terengganu, Perak, and Kelantan [12]. Several publications have identified contributing factors for vaccine hesitancy and refusal that pose challenges in Malaysia (Table 1), as elaborated in the next sections.

**Table 1.** Related publications about vaccine hesitancy in Malaysia

Year	Authors	Title	Methodology	Key findings related to vaccine hesitancy						
				Preference for alternative medicines	Doubt about halal status	Fear of vaccine injury	Concerns about the vaccine’s contents	Conspiracy theories	Convenience and access barriers affecting uptake	Others
2016	Lim et al. [36]	Exploring immunization refusal by parents in the Malaysian context	Non-proportionate stratified random sampling and a structured telephone interview on 39 parents who missed primary immunization for 3 months or longer in government clinics in Kinta, Perak	**√**					**√**	
2016	Azreena Che Abdullah et al. [56]	Practice of childhood immunizations among parents and their associated factors in Hulu Langat, Selangor, Malaysia	A cross-sectional study among 760 parents using cluster random sampling who send their children to selected care centres in Hulu Langat area				**√**			
2017	Ahmad et al. [48]	Primary immunization among children in Malaysia: reasons for incomplete vaccination	A nationwide community-based survey using stratified random sampling design on 10,140 parents who have children aged 12–23 months		**√**				**√**	
2017	Mohd Azizi et al. [50]	Vaccine hesitancy among parents in a multi-ethnic country, Malaysia	A cross-sectional study to understand vaccine hesitancy among 545 parents The study was conducted in the paediatrics and antenatal clinics of a hospital in Kuala Lumpur							Non-Muslim parents more likely
2018	Chan et al. [37]	Trends in vaccination refusal in children under 2 years of age in Kedah, Malaysia: a 4-year review from 2013 to 2016	Data were obtained from the Kedah State Health Department, which covered 60 government health facilities across 11 districts data consisted of information on mothers who were required to sign an opt-out form because they refused either certain or all vaccines for their children below 2 years old	**√**	**√**					
2019	Mohamad Diah et al. [38]	Refusal towards vaccination: a survey among Malay parents	A survey method on 80 Malay parents who have children under 12 years old using a random sampling technique in an urban area	**√**	**√**					
2019	Krishna et al. [51]	Sociodemographic and health care factors in determining immunization defaulters among preschool children in Petaling District, Selangor: a cross-sectional study in Malaysia	A cross-sectional survey among 1,015 mothers with children below 5 years old from 60 registered childcare centres in Petaling, Selangor							Non-Muslim parents more likely
2020	Rumetta et al. [39]	A qualitative study on parents’ reasons and recommendations for childhood vaccination refusal in Malaysia	A qualitative study design using in-depth, face-to-face and online interviews on 14 vaccine refusal parents via purposive sampling and snowballing technique in Klang Valley (urban area)	**√**	**√**	**√**	**√**	**√**		
2020	Kalok et al. [40]	Vaccine hesitancy towards childhood immunization amongst urban pregnant mothers in Malaysia	A cross-sectional study among 1,081 pregnant women who received antenatal care at a teaching hospital in Kuala Lumpur using convenience sampling	**√**		**√**		**√**		Non-Muslim parents more likely
2020	Wong et al. [34]	Vaccine hesitancy and the resurgence of vaccine-preventable diseases: the way forward for Malaysia a Southeast Asian country	A qualitative study design using in-depth, face-to-face, semi-structured interviews with eight health professionals via purposive sampling in a teaching hospital in Kuala Lumpur	**√**	**√**	**√**		**√**		
2023	Ngee Wen and Siti Rohana [49]	Vaccine refusal of National Immunization Program (NIP) in Kedah state	A cross-sectional study where data was collected from a vaccine refusal return form submitted by 64 government health facilities in 11 districts of Kedah from 2016 to 2022		**√**		**√**			

#### Preference for alternative medicines

In a nationwide survey of 6947 respondents, Siti et al. [35] identified a high prevalence of the use of complementary and alternative medicines among healthy people or even chronic disease patients for health maintenance, disease prevention, and treatment in Malaysia. This finding was consistent with several other vaccine hesitancy studies done in the country, which likewise revealed preferences for and strong beliefs in a natural approach, including traditional complementary and alternative medicines such as homeopathy [34, 36-40]. A qualitative study by Wong et al. [34] elaborated how this pocket of vaccine-hesitant people believe the herbal or natural remedies that have been used for generations are safe and could be an alternative to vaccines, even though the evidence of the efficacy of traditional and herbal medicines are generally limited [41]. The study also found that vaccine-hesitant people claim that high uptake of vitamins C and D could cure vaccine-preventable diseases such as diphtheria. In addition, some of this group argue that immunity acquired from a natural infection would provide better and longer protection compared to vaccination [34]. Wan Taib et al. [42] elucidated that there was also a belief that the minerals and vitamins contained in dates, honey, olive oil, and other foods that have been mentioned in the Quran can act as an alternative medicine. A similar reason regarding belief in alternative medicines was also reported among vaccine-hesitant parents in Indonesia [43].

#### Doubt about halal status

Malaysia is a Muslim-majority country, where 61.7% are Muslims [44]. Generally, vaccination is permissible in Islam based on several Islamic legal maxims, such as the preference for the lesser of two harms [45]. Vaccination is also in line with Maqasid al-Shariah, which means the objectives or goals of Islamic law to preserve the five essentials of human well-being: religion, life, intellect, lineage, and wealth. In this case, vaccination is a tool to protect life from a fatal epidemic or pandemic [46, 47]. However, several studies have found that doubt regarding halal status contributes to vaccine hesitancy in Malaysia [34, 37-39, 48, 49]. Two studies conducted in Kedah state are particularly useful in confirming this view by utilizing primary sources of respondents identified by the State Health Department as parents who declined to vaccinate their children and were required to sign the immunization refusal form [37, 49]. Meanwhile, other studies conducted in urban settings have found contradicting results where non-Muslim parents were more likely to be associated with vaccine defaulters [40, 50, 51]. Kalok et al. (2020) suggest that the rulings regarding vaccinations permissible in Islam by the Fatwa Council might have contributed to a comparatively positive impact in terms of compliance among the Muslim population *vis a vis* these other groups [40]. Additionally, Muslims in different nations, including Indonesia and Pakistan, are also concerned about non-halal components in vaccines [52].

#### Fear of vaccine injury

Vaccination is a highly effective way to protect against diseases, but some people may be concerned about the possibility of AEFI. According to the MOH, vaccines are generally safe, but side effects may occur in rare cases [53]. AEFI does not necessarily mean that the vaccine was the cause of the adverse effect. AEFI can happen due to various reasons such as vaccine contents and quality, anxiety during the immunization process, technical errors during preparation or administration, or coincidence events due to unrelated factors [53]. Previous studies have found that one of the reasons for vaccine hesitancy in Malaysia is fear of vaccine injury. For example, a cross-sectional study among 1081 mothers who received antenatal care at a hospital in Kuala Lumpur found that 58% were worried about AEFI [40]. Similarly, a qualitative study found that vaccine-hesitant parents were concerned that vaccines could have negative health effects such as eczema, asthma, autism, and even brain injury [39]. Wong et al. [34] reported that some parents decided not to complete the subsequent vaccination schedule for their children after a perceived AEFI. A study in England also discovered that parents were hesitant to vaccinate their children due to perceived side effects [54]. As AEFI can happen in several situations, it is important to distinguish between coincidental events and genuine vaccine-induced harm; a recently developed WHO tool for AEFI causality assessment can aid in determining the level of certainty of association between an event and the immunization [55].

#### Concerns about the vaccines’ contents

Misconceptions about the safety of vaccine contents also contribute to vaccine hesitancy even though vaccines undergo multiple stages during development, including rigorous phases of testing prior to approval. Ngee Wen and Siti Rohana [49], who conducted a study about vaccine-hesitant parents in Kedah state, identified 26.4% of the respondents rejected vaccines due to the belief that vaccine contents were toxic and impure. A similar finding was also reported by Rumetta et al. [39], in which parents claimed that vaccines contain heavy metal components such as aluminium and mercury that might be detrimental to children’s health. A cross-sectional study by Abdullah et al. [56] on 760 parents in Hulu Langat, Selangor, also found that 1.8% of them did not immunize their children because they thought vaccines were dangerous. A national survey in Italy also revealed that parents hesitated to immunize their children due to doubts with regard to vaccine safety [57].

#### Conspiracy theories

Conspiracy theories have also been identified in the analysis of the reasons behind vaccine hesitancy in Malaysia. According to Rumetta et al. [39], some parents who were hesitant to vaccinate their children believed that pharmaceutical companies and medical doctors were colluding for financial gain. Wong et al. [34] identified a similar scenario, where vaccine-hesitant parents suspected a conspiracy involving vaccine manufacturers, authorities, and doctors. However, both studies had limited sample sizes, and their findings cannot be generalized to the entire country. Kalok et al. [40] also highlighted a lack of trust in the pharmaceutical industry but did not delve into the matter in detail.

#### Convenience and access barriers affecting uptake

The World Health Organization Strategic Advisory Group of Experts on Immunization, in their report in 2014, acknowledged that some parents may have convenience and access issues where they struggle with their busy lives and other pressures, and getting immunizations has become a low priority [6, 58]. There is debate over the classification of these types of issues as ‘vaccine hesitancy’, and today, researchers and governments are encouraged to talk about them as barriers to uptake rather than attributing the failure to vaccinate to the individual [58, 59]. However, these issues have certainly been reported in Malaysia. Lim et al. [36] found that 32.3% of parents who missed primary immunization for 3 months or longer in government clinics in Kinta, Perak, claimed that they were ‘busy at work’ as the reason for missing appointments. This finding was consistent with a study by Ahmad et al. [48], which identified personal reasons such as ‘no time’ or ‘forgotten’ as the cause of incomplete vaccination. Several studies reported that access to the nearest health facilities also impacted the vaccination uptake in Laos [60].

### Initiatives taken by the MOH to optimize access and vaccine acceptance

The MOH monitors closely and takes seriously the vaccine hesitancy threat by establishing a standard operating procedure to manage hesitant parents [12]. Parents are counselled by a medical officer or a family medicine specialist. If the parents refuse to vaccinate, they must sign the opt-out form, reflecting a formal ‘declination’ process [12, 14]. Additionally, the MOH organizes training for vaccine advocates among family health specialists, doctors, and paramedics to equip them with knowledge, including skills, while engaging with parents. Government health facilities also provide immunization kits containing information and FAQs to educate the public. The MOH also employs mass media and social media platforms to disseminate promotion and information about immunization [12, 14]. Furthermore, since 2013, the MOH has collaborated with the Malaysian Paediatric Association as well as the Malaysian Society of Infectious Diseases and Chemotherapy to establish Immunise4Life, an expert-driven community to support the MOH in promoting the NIP to people of all ages [12]. The MOH also works with the Department of Islamic Development Malaysia (JAKIM) to address religious concerns among Muslims [12]. To improve the accuracy of data collection, the MOH is planning an online national immunization registry that will be integrated with the birth registry to track the population. The current immunization record provided to every child is a booklet. Moreover, the MOH is looking to require private clinics to report public health programme data as a condition for registration and licence renewal and is planning to raise awareness about immunization by introducing relevant topics in school syllabi [12].

### Future perspectives

The MOH has implemented and planned positive initiatives for the NIP. The review of the previous studies in light of existing and planned government initiatives gives rise to several further suggestions to further strengthen the implementation of the NIP in Malaysia. For example, the credibility of local leaders, the popularity of influencers or celebrities, and public-private partnerships can be leveraged to promote the importance of vaccination and raise public awareness. Mandates may be an option, subject to the various considerations noted about what responsibilities the state must discharge first. For example, governments must ensure that vaccines are easily accessible, including to citizens who live in remote areas or vulnerable groups. Moreover, health facilities should provide vaccination services without the onerous booking of appointments and long waiting hours. If they are unable to come, the government should reach out to them to offer the service or persuade them [59]. These activities are important given the data showing that access barriers – such as being busy at work – are preventing parents from accessing timely vaccinations for their children. However, countries such as the United States, Italy, France, and Australia have implemented mandatory childhood vaccination requirements for admission to daycares, kindergartens, and schools in part to try and prompt parents to move vaccination up their list of priorities. Some governments go further by using fines: parents in Germany who fail to comply with the vaccination requirements may face a fine of up to €2500 [10]. Therefore, future research should benchmark Malaysia against other countries, especially in the Southeast Asian region, to enhance our understanding of the childhood vaccination landscape. This is attributed to the fact that this review is limited to literature that primarily focuses on the Malaysian context.

## Conclusion

Malaysia has a well-planned and comprehensive approach to implementing, monitoring, and evaluating their NIP. However, vaccine hesitancy is still prevalent due to a preference for alternative medicine, doubts about the halal status of vaccines, fear of vaccine injury, concerns about vaccine contents, conspiracy theories, and convenience and access barriers affecting uptake. The increase in vaccine-preventable diseases and the growing number of vaccine-hesitant parents may prompt the government to consider more coercive vaccination policies alongside other strategies to optimize access and acceptance. However, coercive policies should be pursued with caution to avoid backfiring and should take into account the complex drivers of vaccine uptake.

## Data Availability

Data are available from the cited sources.
